# Rational or altruistic: the impact of social media information exposure on Chinese youth’s willingness to donate blood

**DOI:** 10.3389/fpubh.2024.1359362

**Published:** 2024-05-09

**Authors:** Zhijian Zhang, Qilong Liu

**Affiliations:** School of Journalism and Communication, Huaqiao University, Xiamen, China

**Keywords:** social media exposure to relevant blood donation information, willingness to donate, attitudes toward blood donation, altruism, self-efficacy

## Abstract

**Background:**

Non-remunerated blood donation is the main approach for various medical institutions to get the source of blood supply, but the blood supply shortage is still a problem in today’s society. Social media has become the main approach of information acquisition for youth groups nowadays, and the information on social media will have an impact on people’s behavioral decisions. The objective of this study was therefore to investigate the correlation between social media exposure to relevant information about blood donation and the willingness of youths to donate blood.

**Methods:**

We collected data from 455 questionnaires through an online questionnaire and structural equation modeling was constructed for validation. Data were analyzed for reliability, validity, and demographic differences using IBM-SPSS 26.0, and IBM-SPSS-AMOS 26.0 was used for model fit analysis and path analysis.

**Results:**

The results of the study showed that there was a positive correlation between social media exposure to relevant blood donation information and willingness to donate blood (*β* = 0.262, *p* < 0.001), altruism (*β* = 0.203, *p* < 0.001) and self-efficacy (*β* = 0.170, *p* < 0.001). While there was also a positive correlation between attitude toward blood donation and self-efficacy (*β* = 0.560, *p* < 0.001), there was no positive correlation between it and willingness to donate blood (*β* = −0.180, *p* = 0.786). There was also a positive correlation between altruism and willingness to donate blood (*β* = 0.150, *p* < 0.05) and attitude toward blood donation (*β* = 0.150, *p* < 0.001). Similarly, there was a positive correlation between self-efficacy and willingness to donate blood (*β* = 0.371, *p* < 0.001).

**Conclusion:**

Exposure to more information related to blood donation on social media can increase the willingness of the youth population to donate blood, while exposure to information related to altruism and self-efficacy on social media can also enhance young people’s attitudes toward blood donation, while further strengthening their willingness to donate.

## 1 Introduction

Voluntary, uncompensated blood donation is the primary approach for ensuring the safety and availability of the blood supply and blood products. For maintaining safe and rich blood supply, continuous recruitment and retention of donors are the two essential factors (1). However, according to the World Health Organization, approximately 100 million people donate unpaid blood each year, yet twice as much blood is used as is collected (2).

In China, restrict to the level of people’s cognition, basic medical facilities, policy of blood donation and anti-blood donation psychology, the rate of unpaid blood donation is consistently at a relative low level (1), the statistics of China’s National Health and Health Commission show that the rate of unpaid blood donation has risen from 4.8% in 1998 to 11% in 2017 (3), which only just achieving the goal set by the World Health Organization of 10–20 people per 100 people of unpaid blood donation. The aging trend in China has been increasing in recent years, and the demand for clinical blood has grown dramatically (1). Aging also means that fewer donors will be eligible to donate blood in the future, which will exacerbate the blood shortage. The youth group, as the key group of potential donors, is an important factor in keeping blood donation sustainable. How to guide youth group to receive unpaid blood donation by making incentive policies or information, and increasing the rate of blood donation in the whole society, is a significant part for blood donation career.

In China, social media is an indispensable part of young people’s lives and which is the main ways to obtain information (4). Information from social media is also becoming an important basis for decision-making among young people, guiding their psychology and behavior. With the influence of massive amounts of information, their beliefs and attitudes are gradually changed or strengthened, which in turn affects their behavioral decisions (5). What is the impact of blood donation information on Chinese youth’s willingness to donate blood in the Chinese social media space? How can appropriate social media strategies be adopted to guide and motivate them to donate blood? These questions are important for increasing blood donation rates in China society.

On the other hand, Chinese nations have strong collectivist values and high self-ethical restraints, and the group’s code of conduct and virtue mentality can stimulate the general public’s simple feelings of altruism. Since ancient times, the cultural concept of “man is better than heaven” has been rooted in the hearts of Chinese nations who pay great attention to human mobility and the self-efficacy of individuals in public behaviors. Do these cultural and ethical factors influence the willingness of Chinese youth to donate blood through social media?

The research that has been done on blood donation has focused more on the factors that influence blood donation, such as attitudes, knowledge, social norms. The public health sector is focusing on how to get people to participate in blood donation, and what kind of communication strategies can be developed to increase the rate of blood donation. Less academic research in the past has dealt with the impact of virtue factors on blood donation, such as altruism and empathy, and this study hopes to bring attention to the relationship between virtue factors and blood donation.

The existing research literature on the willingness and behavior of Chinese youth groups to donate blood has paid less attention to the relationship between exposure to social media information and willingness to donate blood, but social media has become the main source of information acquisition for contemporary Chinese youth groups, this information obtained from social media also greatly influences the decision-making of youth groups. Secondly, there are both theoretical models for the prediction of blood donation intention and behavior, mainly the theory of planned behavior (6–8) and the theory of rational behavior (9), the theory of rational behavior can be used for predicting and explaining individual’s behavior, which contains variables such as beliefs, behavioral attitudes, subjective norms, behavioral intentions and actual behaviors, and believes that people’s behaviors are made in the context of rational thinking; the theory of planned behavior is based on the theory of rational behavior to add the variable of perceptual behavioral control to better predict individual’s behavior. The theory of planned behavior is based on the rational behavior theory by adding the variable of perceived behavioral control, which can better predict individual behavior. Those theoretical models have a certain degree of explanatory and predictive power, and they emphasize the influence of social pressure and self-anxiety on the willingness to donate blood, but they do not give more consideration to the virtue factor driven by altruism, so they cannot predict or explain behaviors that include the virtue factor. And as a public good behavior, altruism based on virtues is also an extremely important part, which plays an important role in the blood donation activities of medical institutions.

Based on the above background, this study introduces youth’s social media information exposure, altruism, attitude toward blood donation, self-efficacy, and willingness to donate blood as important variables into the study, and also introduces the behavioral willingness variable in the Theory of Reasoned Acts, extends the Theory of Reasoned Acts model (TRA). An attempt was made to integrate the construction of structural equation modeling to validate the influence paths between Chinese youth’s social media information exposure and their willingness to donate blood, to explore what are the important factors influencing their willingness to donate blood, to further explain and predict their willingness to donate blood, and to explore the social media communication strategies to improve Chinese youth’s willingness to donate blood on this basis.

## 2 Literature review and hypothesis

### 2.1 Access to blood donation related information

Social media platforms have become the main source of information acquisition for contemporary youth groups, who are often actively or passively exposed to information related to blood donation when browsing social media. The development of social media platforms and the popularization of electronic information technology, which also includes the increased willingness of individuals to self-expression, have further influenced the exposure of social media information, which has had a different impact on the users’ behaviors and attitudes (10). Gavgani’s study showed that college students self-assess their health status and seek health information on online platforms (11), that gathering health information changes their assessment of their own health status and influences perceptions of diseases. Existing research suggests that people’s active and passive exposure to health-related information informs their health decisions and influences their lifestyle habits (10, 12).

Some studies have discussed the relationship between exposure to blood donation information on social media and willingness to donate. Agrawal’s study found (13) that the increase and marketing of blood donation content in the electronic media can increase blood donations across a state or region, implying that exposure to media messages can enhance attitudes and increase willingness to donate blood. In addition, Dabula’s study on millennials (14) supports this finding. Rabeeh and Onaiza (15) et al. pointed out that voluntary blood donation messages forwarded on YouTube are delivered online and the virtue factor promotes the users to go to the blood stations to donate blood, in addition to the study of Alanzi and Alfayez (16) confirms this.

Lin (17) and Shah (18) illustrated in their study that media exposure behaviors can have a positive or negative effect on the audience. In a study of cancer patients in the United States (19), it was found that patients who received more information about health treatments on the Internet increased their self-efficacy and shifted their attitudes toward the disease, suggesting that there is a positive correlation between exposure to health information and self-efficacy, which also implies that people who are exposed to more information about blood donation have the potential to increase their attitudes toward and self-efficacy toward donating blood. Based on this, we propose the following hypothesis:

*H*1: Social media exposure to information related to blood donation has a positive impact on attitudes toward blood donation in the youth population.

*H*2: Social media exposure to information related to blood donation has a positive impact on willingness to donate blood in the youth population.

*H*3: Social media exposure to information related to blood donation has a positive impact on altruism in the youth population.

*H*4: Social media exposure to information related to blood donation has a positive impact on self-efficacy in a youth population.

### 2.2 Theory of rational behavior

Theory of Reasoned Action (TRA) is a well-known attitude-behavior relationship theory in social psychology, which is mostly used to explain and predict individual intentions and behaviors (20). In 1963, Fishbein and Ajzen proposed the multi-attribute attitude model (21), which argues that behavioral attitudes determine behavioral intentions. In 1975 Fishbein and Ajzen proposed the theory of rational behavior (22), which further improved the model on the basis of the original theory and added the factor of subjective norms, which argues that individual behavior is influenced by behavioral intentions, and human behavior is in turn influenced by social external pressures such as attitudes and behavioral norms.

In the field of health research, the theory of rational behavior is an important classical framework to explaining and analyzing individual health behaviors, and the theory of rational behavior has good explanatory and predictive power for individual-specific behavioral intentions and behavioral decisions. In existing studies, the theory of planned behavior is mostly found in the management of smoking surveys (23), consumer psychoanalysis (24), psychoanalysis (25), infectious disease control (26), health screening (27), health information sharing (28), vaccination (29, 30), and neocoronavirus pneumonia (31, 32) studies, etc.

In a survey of medical students, Chauhan and Kumar (33), etc., noted a positive correlation between attitudes toward blood donation and willingness to donate, there was also a positive correlation between unpaid donors who hold positive blood donation attitudes and donating blood again. Predictive analyses of Australian blood donors showed (34) that there was a positive correlation between attitudes toward blood donation and self-efficacy, which was also proved by Liu’s study (35), this implies that when people have a more positive attitude toward donating blood, their self-efficacy and willingness to donate may also be stronger. Based on which the following hypothesis was proposed:

*H*5: Attitudes about blood donation have a positive impact on willingness to donate in the youth population.

*H*6: Attitudes about blood donation have a positive impact on self-efficacy in youth groups.

### 2.3 Altruism

Altruism refers to a conscious, voluntary behavior beneficial to others (36), which is not motivated by the expectation of compensation for external benefits or avoiding external punishment, but by moral self-awareness. Essentially a self-sacrificing and moral ideal, altruism can help individuals and provide motivational support (37). A questionnaire is used to measure people’s altruism, such as “Donating blood to help others brings personal satisfaction,” which contains six items to measure people’s level of altruism. Altruism has been shown to play a role in people’s daily life decisions, for example, Sojka (37) stated that altruism is a common reason for continuing to donate blood among unpaid donors, and that there is a correlation between altruism and the willingness of donors to re-donate blood behaviors. This implies increased altruism, which may lead to increased willingness to donate blood. And in a survey of Iranian population (38), Mousavi stated that altruism plays a dominant role in positive attitudes toward unpaid blood donation. The implication is that people who are more altruistic are likely to have a more positive attitude toward blood donation.

Based on this, the following assumptions are made:

*H*7: Altruism has a positive effect on attitudes toward blood donation in youth groups.

*H*8: Altruism has a positive effect on the willingness to donate blood in youth groups.

### 2.4 Self-efficacy

The concept of self-efficacy (SE) was developed by Bandura and refers to an individual’s beliefs about the ability of the self to achieve specific behaviors (39). Self-efficacy can affect an individual’s behavior choice and willingness (40). Self-efficacy has been found to be significantly related to individuals’ behavioral decisions, beliefs and intentions, and has been found to have an impact on health issues such as diabetes (41, 42) and vaccinations (43, 44). In a survey of Australian residents, Clowes and Masser (45) showed a positive correlation between self-efficacy and willingness to donate blood. In Liu’s research on Chinese university students’ willingness to donate blood (35), a significant association was found between self-efficacy and attitudes toward blood donation and willingness to donate blood. This means that those with a higher sense of self-efficacy may have a stronger willingness to donate blood.

Based on this, the following assumptions are made:

*H*9: Self-efficacy has a positive effect on willingness to donate blood in youth groups.

### 2.5 Willingness to donate blood

Willingness to donate blood refers to how likely an individual is subjectively to engage in the act of donating blood. Existing research on willingness to donate blood is mainly based on the theory of planned behavior, which contains factors such as behavioral norms, perceived control, self-efficacy, attitude, blood donation anxiety, and so on (46–48). While the consideration of social media information exposure and moral dimension is insufficient, there is a lack of research on blood donation intention and social media exposure. Liu (34) and Lam (49) studied the consideration of moral factors on blood donation intention, while Helen was more interested in studying the influence of media communication and family on blood donation intention (14). Therefore, this study is a combination of social media information exposure and moral altruistic considerations to predict and analyze the willingness to donate blood in the youth population.

## 3 Methods

### 3.1 Participants

This study focuses on the impact of exposure to blood donation-related information on social media platforms on the willingness of Chinese youth groups to donate blood. According to China’s blood donation law (50), the age requirement for blood donors is 18–55 years old, and one of “China’s Medium- and Long-Term Youth Development Plans” (51) states that the range of youth is 14–35 years old. Therefore, the participants in this study were the youth group aged 18–35 years old.

### 3.2 Data collection

This study adopts the method of online random questionnaire survey, relying on the current China’s largest online questionnaire survey platform—Questionnaire Star^1^ to produce and generate questionnaires, and distributing and collecting questionnaires through online social media platforms such as WeChat, QQ, Douban, and Weibo, etc. Under the premise of guaranteeing that the user’s privacy is strictly protected, the data collection is divided into two stages: the pre-survey and the formal survey.

The first stage was a pre-survey, which was conducted to help users better understand the questions in the formal survey phase, as well as to measure the reliability and validity of the questionnaire. This phase started on August 26, 2023, and a total of 92 questionnaires were collected through Questionnaire Star, with 85 valid questionnaires, and the questionnaire was revised based on the results of the reliability and validity.

The second stage was the formal survey, which began on September 6, 2023 and ended on September 28, 2023, a total of 593 questionnaires were collected in this stage, excluding invalid samples (too short answer time, the same IP address repeated, etc.) and 138 questionnaires that did not meet the requirements of the participants in this study (age does not meet the age of 18–35 years old), and finally obtained a valid questionnaire 455 questionnaires (*n* = 455), the questionnaire The effective rate of collection was 76.7%.

The demographic characteristics of the participants are shown in Table 1. Of the 455 valid participants, the largest percentage of age was 18–24 years old at 59.1%, followed by 25–34 years old at 40.9%. In terms of educational background, most of the participants have bachelor’s degree or above, including 1.98% with less than bachelor’s degree, 51.87% with bachelor’s degree, 43.96% with master’s degree, and 2.2% with doctoral degree or above. In terms of monthly income, the largest proportion of monthly income <3,000 was 56%, monthly income in 3,000–5,999 accounted for 20.4%, in 6,000–9,999 accounted for 14.3%, and monthly income ≥10,000 accounted for 9.2%.

**Table 1:** Demographic characteristics of participants and Differential testing of demographic characteristics in behavior (*N* = 455).

Characteristic	Demographic information	Frequency	%	Mean	SD	t, F, or r	*P*-value
Gender*	Male	134	29.4	1.71	0.456	0.247	0.769
	Female	321	70.6				
Age	18–24	269	59.1	2.41	0.492	2.491	0.115
	25–34	186	40.9				
Education	Primary school and below	2	0.4	4.45	0.634	0.243	0.962
	Junior High School	2	0.4				
	Senior High School	5	1.1				
	Bachelor college	236	51.9				
	Master’s degree	200	44				
	Doctoral degree and above	10	2.2				
Monthly Income (CNY)	<3,000	255	56	1.77	1.01	0.632	0.595
	3,000–5,999	93	20.4				
	6,000–9,999	65	14.3				
	≥10,000	42	9.3				

Variables with “*” indicate the heterogeneity of variance, using the Welch method. SD is the standard deviation. “CNY” refers to the China Yuan.

### 3.3 Measures

The questionnaire of this study consisted of 6 parts, which were participants’ demographic and sociological data, social media exposure, and attitude toward blood donation, altruism, self-efficacy, and willingness to donate blood. The independent variable items were measured using 5- and 7-point Likert scales, and to ensure the accuracy of the questionnaire, a strict translation-back-translation procedure of the questionnaire was carried out in this study, whereby the English scales were translated into Chinese and then back-translated to ensure that the statements were accurately stated and free of ambiguity.

#### 3.3.1 Social media exposure

Measurement of Social Media Exposure to Information about Blood Donation, adapted from Mars, Donovan (52), and Pan (53), among others, measured social media exposure to information about blood donation in a youth population using three questions, with sample questions including “Have you ever been exposed to information about blood donation on social media platforms such as microblogging and Wechat?,” “Have you ever searched for information about blood donation on social media platforms such as microblogging, Xiaohongshu, Douban, etc.,” “Have you ever communicated with others on topics and content about blood donation on social media platforms such as WeChat and microblogging?. The question items were all measured on a 5-point Likert scale, (1 = No, 2 = Less, 3 = Fair, 4 = More, 5 = Lots) Cronbach’s coefficients was excellent (α = 0.852, M = 2.7392, SD = 1.13022).

#### 3.3.2 Attitudes toward blood donation

The measurement of attitudes toward blood donation in this study was adapted from the study of Christopher (54) and Masser (55) etc. Three questions were used to measure the attitudes of the youth population toward blood donation, sample questions included, “I think that the act of donating blood without compensation is good,” “I think blood donation is enjoyable,” “I think blood donation is rewarding.” and the items were measured on a 5-point Likert scale, (1 = strongly disagree, 2 = partially disagree, 3 = not sure, 4 = partially agree, 5 = strongly agree), with excellent Cronbach’s coefficients (α = 0.868, M = 3.9927, SD = 0.88383).

#### 3.3.3 Altruism

Altruism is primarily defined as the motivation for intentional, voluntary behavior to help others in everyday life. For the measurement of the altruism variable, it was adapted from Martin’s (56) study to measure altruism with six questions, example question: “‘Donating blood can help someone or save a life and contribute to human solidarity,” “Donating blood to help others can bring personal satisfaction,” “Blood cannot be produced, we need to work together,” “Donating blood makes me feel that I am valuable and useful to society “, “Donating blood is fulfilling a social and moral obligation to help others,” “Donating blood does not require much effort,” the questions were all measured using a 5-point Likert scale, (1 = strongly disagree, 2 = partially disagree, 3 = not sure, 4 = partially agree, 5 = strongly agree), with excellent Cronbach’s coefficients (α = 0.886, M = 4.0033, SD = 0.76428).

#### 3.3.4 Self-efficacy

Self-efficacy refers to the ability of an individual to be able to overcome external obstacles to make something happen, this paper refers to the study by Clowes and Masser et al. (45) to measure self-efficacy with five questions, sample question: “I believe there are things I can do to avoid a bad blood donation experience,” “I am able to reduce the intensity of negative reactions to blood donation, such as dizziness, weakness, or nausea,” “There are steps I can take to minimize uncomfortable reactions to donating blood,” “There are things I can do prior to donating blood that will increase the positive psychological experience of donating blood,” and “There are steps I can take to control the effects of negative emotions on me,” the questions were all measured using a seven-point Likert scale to measure, (1 = strongly disagree, 2 = disagree, 3 = partially disagree 4 = not sure, 5 = partially agree, 6 = agree, 7 = strongly agree), with excellent Cronbach’s coefficients (α = 0.913, M = 4.8066, SD = 1.33237).

#### 3.3.5 Willingness to donate blood

The variable about willingness to donate blood was adapted from Kowalsky (57) and Clowes (45) etc. to measure people’s willingness to donate blood with three question items, example question: “In the next 3 months, I have the intention to go and donate blood,” “How likely am I to go and donate blood in the next three months,” “In the next 3 months, I have decided to go and donate blood “, the questions were all measured on a 5-point Richter scale, (1 = strongly disagree, 2 = partially disagree, 3 = not sure, 4 = partially agree, 5 = strongly agree), with excellent Cronbach’s coefficients (α = 0.955, M = 2.5311, SD = 1.24779).

### 3.4 Data analysis methods

The study used IBM-SPSS 26.0 and IBM-SPSS-AMOS 26.0 (Statistical Package for the Social Sciences and IBM-SPSS-AMOS 26 software packages) to process and analyze the data. Descriptive statistical analysis, reliability analysis, Pearson correlation analysis, independent samples t-test, one-way ANOVA (one-way ANOVA), and exploratory factor analysis were performed using SPSS.AMOS was used for the validation factor analysis (CFA), structural equation goodness of fit test, and path coefficient (β), R^2^, f^2^, and Q^2^.

## 4 Data analysis result

### 4.1 Differential testing of demographic characteristics

In order to investigate the differences in willingness to donate blood by different demographic characteristics, this study was conducted using one-way ANOVA using SPSS. The premise of one-way ANOVA is to ensure that the independent groups are homogeneous, so the data need to be tested for homogeneity of variance. If the *p*-value is less than 0.05, it means that the variance is not homogeneous, and if the p-value is more than 0.05, the ANOVA test result will be the final result (as shown in Table 1). The results showed that there was no significant difference between the control variables such as gender, age, education, and income in terms of willingness to donate blood.

### 4.2 Correlation analysis of each variable

In order to measure the relationship between the variables, we used Pearson’s correlation to analyze the correlations between blood donation attitude, social media exposure, altruism, and self-efficacy. The results of the analysis showed that there was a significant positive correlation between social media exposure and blood donation attitude, altruism, self-efficacy, and willingness to donate blood (*p* < 0.01); a significant positive correlation between altruism and blood donation attitude, self-efficacy, and willingness to donate blood (*p* < 0.01); a significant positive correlation between blood donation attitude and self-efficacy, and willingness to donate blood (*p* < 0.01); and self-efficacy and willingness to donate blood showed a significant positive correlation (*p* < 0.01) between self-efficacy and willingness to donate blood. The results of the analysis also verified the relationship between the relevant variables of the hypothesized model (as shown in Table 2).

**Table 2:** Correlation analysis between different variables.

	Information exposure	Altruism	Attitude	Self-efficiency	Intention	M	SD
Information Exposure	1					2.739	1.130
Altruism	0.203**	1				4.003	0.764
Attitude	0.210**	0.762**	1			3.344	0.826
Self-efficiency	0.390**	0.673**	0.596**	1		4.807	1.332
Intention	0.288**	0.434**	0.368**	0.530**	1	2.531	1.24876

^**^P < 0.01.

### 4.3 Confirmatory factor analysis

In order to measure the variables in the model of this study, this study refers to the proven scales with full reference to the existing studies, which were slightly adapted to apply to the scope of this study and validated by the method of validation factor (CFA) analysis. Exploratory factor analysis of all scale questions using SPSS 26.0 showed that the KMO value for the content of the questionnaire was 0.923 (>0.6), and the Bartlett’s spherical test *p* = 0.000 (<0.05), indicating that the questionnaire was well suited for factor analysis. Principal component analysis and Kaiser normalized maximum variance method were used with 6 rotations, of which 5 items had eigenvalues greater than 1. The cumulative variance contribution was 73.3% (>60%).

**Figure 1:**
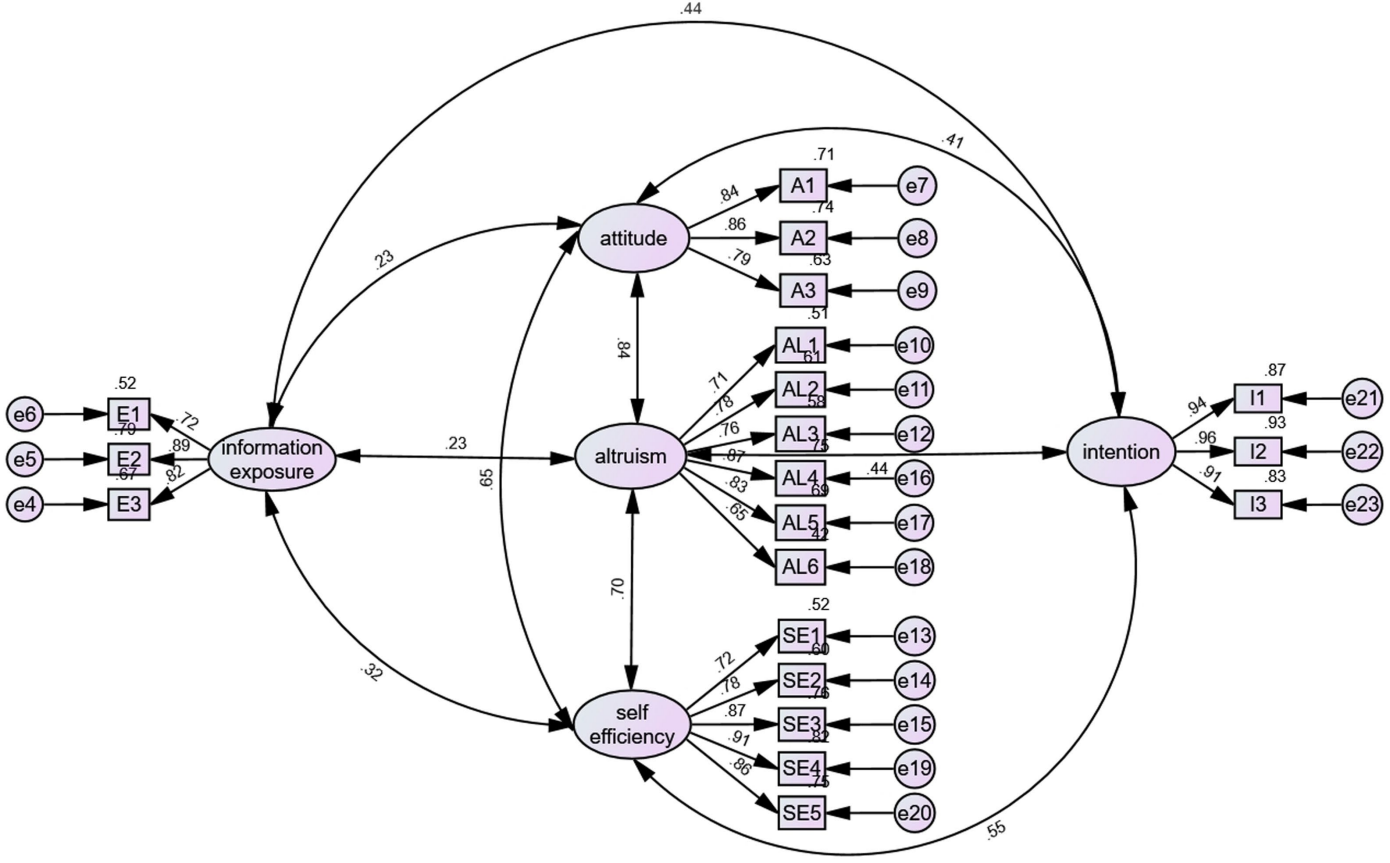
Confirmatory factor analysis.

**Figure 2:**
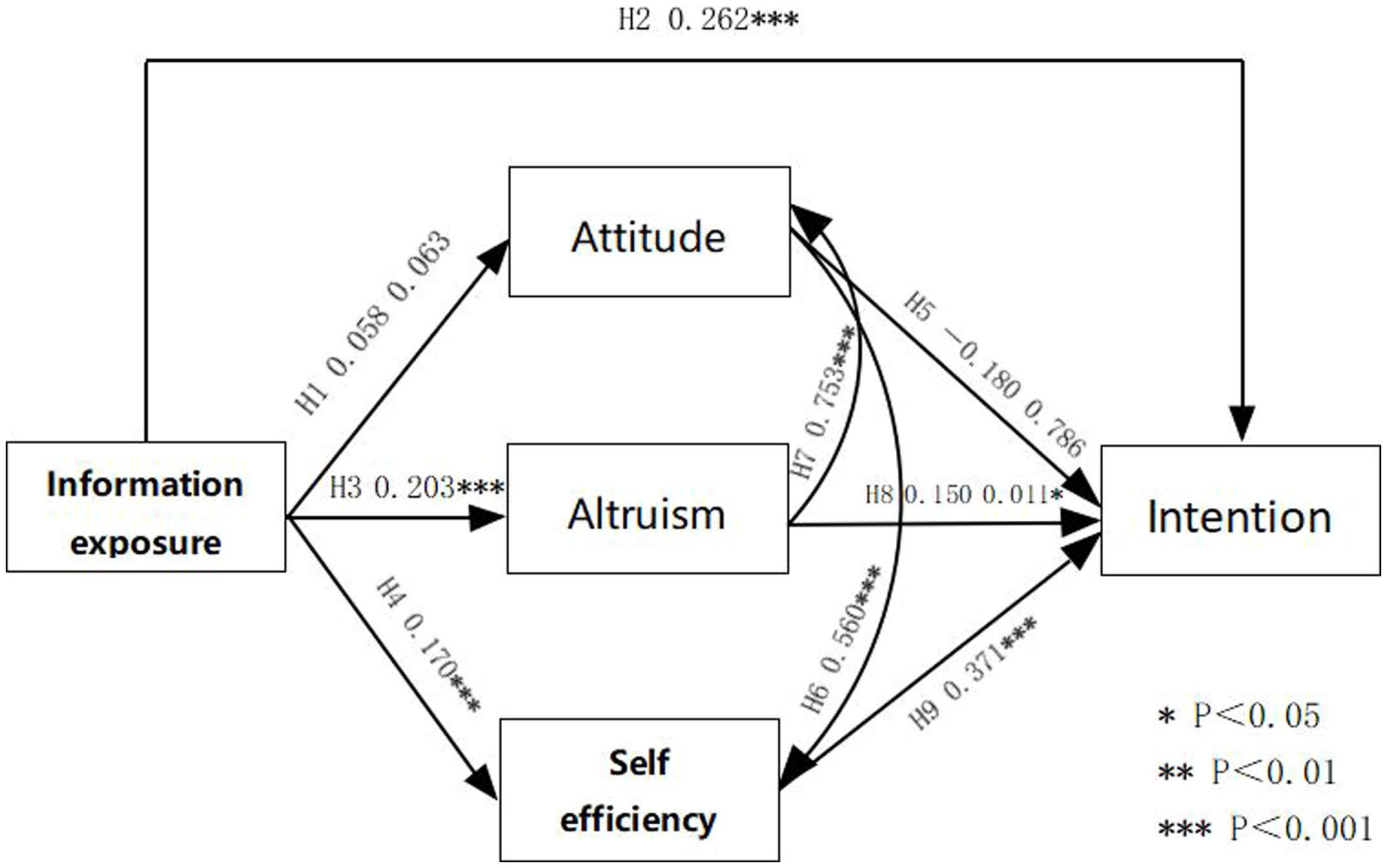
Research model.

The questions were subjected to validation factor analysis using AMOS 26.0, which is the process of verifying the consistency between the hypothesized structural model and the actual data. The results showed (as shown in Figure 1) that the factor loading coefficient coefficients for each question item ranged from 0.722–0.964, which were above the threshold of 0.5 (as shown in Table 3), which indicates high correlation and high convergent validity of the measures of each latent variable and suggests that the questionnaire is more reasonable in terms of the questions it sets (Figure 2).

**Table 3:** Factor loadings.

Variables	Item 1	Item 2	Item 3	Item 4	Item 5	Item 6
Information exposure	0.725	0.889	0.818	/	/	/
Intention	0.935	0.964	0.909	/	/	/
Attitude	0.836	0.878	0.774	/	/	/
Self-efficiency	0.734	0.814	0.891	0.865	0.812	/
Altruism	0.722	0.794	0.77	0.868	0.83	0.879

### 4.4 Reliability and validity testing

In this study, Cronbach’s alpha was used as an evaluation criterion for the reliability of the measurement question items. The Cronbach’s coefficient values of the variables were shown in Table 4 to be between 0.852 and 0.955, with a reliability coefficient value of >0.8.According to Fornellhe Larcker (58), the study showed that alpha values of >0.7 are more reasonable at the time of the questions, indicating that the measurement items in this study have very good reliability, as well as good internal consistency.

**Table 4:** Reliability and convergence validity testing results.

Latent variables	Cronbach’s Alpha	AVE	CR
Information exposure	0.852	0.662	0.854
Intention	0.955	0.877	0.955
Attitude	0.868	0.690	0.869
Self-efficiency	0.913	0.681	0.914
Altruism	0.886	0.660	0.921

CR is the composite reliability; AVE is the average variance extracted, Cronbach’s Alpha is the extent to which the items represent the scale.

The validity test of the scale mainly contains three parts, content validity, convergent validity and discriminant validity. In terms of content validity, this study draws on existing mature scales as well as a large number of previous studies, and shows better results in the pre-testing process, indicating that the content validity of this study is more reasonable and guaranteed.

At the level of convergent validity, in this study, according to the results in Tables 3, 4, the factor loading coefficient estimate for each question item is greater than 0.5, which satisfies the range criterion of Fornellhe (58), and the combined reliability (CR) of each latent variable is greater than 0.7, as well as the average variance extracted value (AVE) is greater than 0.5.This indicates that convergent validity of all the question items in this study is Good.

In terms of discriminant validity, according to the results in Table 5, the correlation coefficients between the latent variables are less than the square root of AVE, which indicates that the discriminant validity of all the measurement items in this study is good. To summarize, the questionnaire in this study is well designed and the items themselves have good reliability and validity.

**Table 5:** Discriminant validity testing results.

	Information exposure	Altruism	Attitude	Self-efficiency	Intention
Information Exposure	**0.813**				
Altruism	0.208***	**0.812**			
Attitude	0.230***	0.787***	**0.830**		
Self-efficiency	0.321***	0.688***	0.653***	**0.829**	
Intention	0.437***	0.467***	0.414***	0.562***	**0.936**

The bold values indicate the square root of AVE. *Indicate the heterogeneity of variance, using the Welch ANOVA.

### 4.5 Model fitting

In this study, the structural equation model was constructed using Amos 26 and its fit was assessed using the following suggested criteria for model fit: (1) relative chi-square (x2/df), which should be between 1 and 5; (2) root mean square of approximation error (RMSEA), which should be less than 0.08; (1) (NFI), which should be greater than 0.9; (2) Tucker-Lewis Index (TLI), should be greater than 0.9; (3) Comparative Fit Index (CFI), should be greater than 0.9.

The initial model was evaluated and the results showed that the value of x2/df was 3.542, the value of RMSEA was 0.081, the value of NFI was 0.883, the value of TLI was 0.913, and the value of CFI was 0.926. The RMSEA and NFI indicators did not meet the recommended criteria, the initial model was not very satisfactory, and it needed to be corrected. Therefore, according to the modified indicators Modification Indices (MI) output from the initial model in Amos, the covariate relationships were established to reduce the chi-square values and further fit the model. The modified model indices are shown in Table 6: the value of x^2^/df is 3.176, the value of RMSEA is 0.069, the value of NFI is 0.914, the value of TLI is 0.942, and the value of CFI is 0.952. All the modified model indices are in line with the fitness indexes, and the model is well fitted (as shown in Table 6).

**Table 6:** Model fitting indexes after modification.

Index	x^2^/df	RMSEA	CFI	TLI	NFI
Observed value	3.176	0.069	0.952	0.942	0.914
Ideal value	<5	<0.08	>0.9	>0.9	>0.9

RMSEA is the root means square error of approximation; NFl, Normed fit index; TLI, Tucker-Lewis index; CFl, comparative fit index.

### 4.6 Hypothesis testing

Nine paths of the structural equation modeling of this study were analyzed using Amos26. The results of the path coefficient plots and hypothesis testing analyses are shown in Table 7 and FINGURE2. *p*-values for the 7 paths, H2, H3, H4, H6, H7, H8, H9, were < 0.05, suggesting that the 7 paths were connected. There was a positive correlation between social media information exposure and willingness to donate blood, indicating support for H2 (*β* = 0.262, *p* < 0.001); between social media information exposure and altruism (*β* = 0.203, *p* = 0.146), indicating support for H3; and between social media information exposure and self-efficacy (*β* = 0.170, *p* < 0.001), indicating that H4 was supported; a positive correlation between attitude to donate blood and self-efficacy (*β* = 0.560, *p* < 0.001), indicating that H6 was supported; a positive correlation between altruism and attitude to donate blood (*β* = 0.753, *p* < 0.001), indicating that H7 was supported; a positive correlation between altruism and willingness to donate blood (*β* = 0.150, *p* = 0.011), indicating support for H8; and a positive correlation between self-efficacy and willingness to donate blood (*β* = 0.371, *p* < 0.001), indicating support for H9.

**Table 7:** Hypothesis testing results.

Hypothesis	Model paths	Path coefficients(β)	*p*-values	Results
H1	Information exposure→Attitude	0.058	0.063	Not supported
H2	Information exposure →Intention	0.262	***	Supported
H3	Information exposure→Altruism	0.203	***	Supported
H4	Information exposure→Self-efficiency	0.170	***	Supported
H5	Attitude→ Intention	−0.180	0.786	Not supported
H6	Attitude→Self-efficiency	0.560	***	Supported
H7	Altruism→Attitude	0.753	***	Supported
H8	Altruism→Intention	0.150	0.011*	Supported
H9	Self-efficiency→Intention	0.371	***	Supported

*Indicate the heterogeneity of variance, using the Welch ANOVA.

In summary, H2, H3, H4, H6, H7, H8, H9 are supported.

## 5 Discussion

### 5.1 Influence on social media about blood donation information exposure and blood donation attitudes, willingness, altruism, and self-efficacy

The study found a positive correlation between exposure to blood donation information on social media and willingness to donate in the Chinese youth population, which is also in line with a previous study by Duh et al. (49). This stems from the fact that social media is a platform with high information interactivity, which allows for the rapid dissemination of information about blood donation on social platforms. In addition, there is also a positive correlation between information exposure and self-efficacy of the youth group, which stems from the fact that the sharing of blood donation feelings on social media platforms is skewed toward positivity, which has a positive relationship with people’s self-efficacy, and corroborates Ortiz’s (59) study that when the source of the information is reliable they are also more interested in getting involved in the matter. In addition, there is a positive correlation between exposure to relevant blood donation information and people’s altruism, this is because the attitude of the information affects the formation of people’s altruism, and Soylu’s study also proves that there is a positive relationship between positive attitudes and altruism when it comes to positive attitudes and altruism (60).

Contrary to the research hypothesis, exposure to social media-related blood donation information did not have a significant positive correlation with attitudes toward blood donation, which may stem from the fact that attitude formation is a combination of multiple factors, for example, Crano’s (61) study found that the formation of blood donation attitudes also includes the influence on groups such as family and friends. As well as the echo chamber formed by the information cocoon under social media also affects attitudes, selective exposure of an individual is not something that will change an individual’s attitudes, but sometimes reinforces the original attitudes, which is why there is no positive correlation between social media information exposure and attitudes toward blood donation. In addition, the credibility of the source, the type of information, and the proliferation of invalid information and rumors may also account for the lack of a positive correlation between exposure to information and attitudes toward blood donation.

### 5.2 Influence on attitudes and willingness to donate blood, self-efficacy

An unexpected finding was that there was no significant positive correlation between attitude toward blood donation and willingness to donate, a finding that is different from established studies (62–64), which demonstrated a positive correlation between positive attitude toward blood donation and willingness to donate, but this study found no significant positive relationship between attitude and willingness to donate. It was hypothesized that there are other factors in the process of attitude to behavioral willingness to donate, such as fear of donating blood (65), anxiety about donating blood (66), anticipatory emotions (67), and the environment of donating blood (68), which are important factors affecting the public’s willingness to donate blood. Masser (45) demonstrated that there is a positive relevant relationship between attitude toward donating blood and self-efficacy, which is consistent with the hypotheses of the present study. This suggests that existing blood donation campaigns should pay more attention on enhancing people’s self-efficacy, including upgrading the environment and medical blood collection equipment at blood stations, which can promote people to donate blood.

### 5.3 The influence on altruism and attitudes toward blood donation and willingness to donate blood

Altruism has been shown to have a positive correlation between attitudes toward blood donation and willingness to donate, for example, Evans and Scholz (69, 70), which also shows that altruism plays an important role as a moral factor in the process of blood donation. China has a profound culture of benevolence, love and morality, and the concepts of collectivism and self-giving are more prevalent. The general public pays great attention to the individual’s moral influence and sense of social responsibility in the society, and donating blood is an event for them to show their self-giving spirit, and it can demonstrate their self-giving spirit. In China, where collectivism is prevalent, altruism is often the root and source of self-giving, so increased willingness to donate blood is often related to altruism. This is different from previous blood donation campaigns, which focused more on the harmlessness of blood donation and less on the altruism of the recipients, which may be one of the reasons for the lack of participation in blood donation. In view of the above, healthcare organizations need to make full use of China’s strong atmosphere of collectivism to strengthen the promotion of ethical blood donation to the people.

### 5.4 Effect of self-efficacy on willingness to donate blood

Fear and anxiety on blood donation in the process of blood donation can make some people more rejected to donate blood, which will also affect their attitude and willingness to donate blood (71). Through the publicity of blood donation knowledge and related psychological explanations and guidance, the fear and anxiety of blood donation can be alleviated, at the same time, the level of the existing blood donation environment and equipment has been gradually improved that can change people’s view on blood donation, which has also improved their self-efficacy in the process of blood donation. However, the existing promotion of self-efficacy in blood donation is still insufficient, and medical blood donation institutions can mobilize and strengthen people’s self-efficacy and spiritual attributes to increase the rate of blood donation among young people.

### 5.5 The relationship between blood donation attitudes, altruism and self-efficacy in information exposure and willingness to donate blood

The study found a positive correlation between exposure to social media information about blood donation and altruism, as well as a positive correlation between both exposure to information and altruism and willingness to donate, implying that increasing the level of exposure to information about altruism increases willingness to donate, suggesting that there may be a mediating role for altruism in the relationship between exposure to information and willingness to donate blood. Similarly, self-efficacy may have a mediating role between information exposure and willingness to donate blood. Unexpectedly, there is a positive correlation between attitude toward blood donation and exposure to social media information about blood donation, but there is no positive correlation between attitude toward blood donation and willingness to donate, meaning that increasing the level of attitude toward exposure to information will not increase people’s willingness to donate, which we guess may be due to the attitudinal bias of the source and the message, Hyde’s research has also found that whether the source and the message is positive or not will affect people’s attitude toward the things, and then affect the willingness to act (72). That also prompts us to focus on the authority and positivity of the content as well as the influence and shaping of the cultural environment on people’s thinking in future blood donation campaigns (73), while measures can be taken to strengthen people’s will as a way to reduce the paradox between attitudes and willingness (74). In addition, we found a positive correlation between altruism and attitudes, and a positive correlation between blood donation attitudes and self-efficacy, implying that attitudes may have a mediating role between altruism and self-efficacy, which is consistent with previous studies (34, 75).

## 6 Limitations

This study still has a bit of limitations and needs more refinement in the future. Firstly, the different types of information about blood donation on social media were not segmented, as well as examining information from different sources, Variations in the type of information and differences in the sources can have an impact on attitudes and intentions, and therefore the results may not be sufficiently relevant or biased. Secondly, established studies have found that there are other factors influencing the relationship between attitudes and willingness to donate, which means that more variables could be introduced into this for study, such as blood donation anxiety, fear of donating, anticipatory emotions and other factors, which have not been studied in more depth due to financial support. Thirdly, this study only examined altruism, which also includes pure and impure altruism, and in the context of collectivism in China, it seems that there is a need for a separate study of pure and impure altruism. Fourthly, the research methodology of this study is retrospective, which means that there will be bias in the process of investigation and this will affect the final results, which can be investigated in future research in the form of thought-probe. Fifthly, it was found during the research that will may play a role between attitude and behavior, but based on the research purpose of this paper did not do a more in-depth step to discuss this, which can be explored in more depth in future research.

## 7 Recommendations

It is important to develop communication strategies that promote altruism, self-efficacy and willingness to donate blood on social media platforms. This study demonstrates that self-efficacy and altruism are important factors in increasing willingness to donate blood, which has practical implications for blood donation work and volunteer recruitment, and can provide a basis for professional staff to develop more detailed and appropriate communication strategies, for example, the relevant blood donation organizations to enhance the promotion of altruism and morality, and the social media information campaigns to focus on stimulating the sense of morality and efficacy, as well as the production of relevant short films and videos for dissemination, as well as post-donation supplementation and psychological counseling to help people to donate. There is also a need to produce relevant short films and videos for dissemination, as well as nutritional supplements and psychological counseling after blood donation, to help people establish a correct understanding, so as to increase the willingness of young people in the whole society to donate blood and save more lives. In addition, medical institutions need to provide scientific and correct guidance to Internet users’ discussions and understanding of blood donation to reduce misinterpretation and stigmatization of blood donation. And public policy makers need to take full advantage of the social atmosphere of altruism, enhance the advocacy of altruistic behavior, and cultivate positive social attitudes. Finally, in-depth interviews or thought probe measurements can be adopted in future research to minimize bias while giving more depth to the study. The results of this study have important practical implications for the development of public health strategies in China.

## 8 Conclusion

Against the backdrop of a major “blood panic” in major hospitals and medical institutions, this study aims to investigate the effects of social media exposure to relevant blood donation information, attitudes toward blood donation, altruism, and self-efficacy on the willingness to donate blood in a Chinese youth population. On the basis of rational behavior theory, we constructed a structural equation model that includes rational behavior theory, self-efficacy and altruism to predict and explain people’s willingness to donate blood, and we extended the rational theory model and introduced altruistic variables to predict people’s willingness to donate blood. Through the questionnaire survey and model testing we found that the more frequent the exposure to blood donation related information in the youth group, self-efficacy and altruism will be stimulated, and altruism and self-efficacy have a positive effect on people’s willingness to donate blood, so the study found that self-efficacy and altruism are important factors to increase people’s willingness to donate blood. This study also found that there may be complex mediating effects between variables, such as blood donation attitude, altruism and self-efficacy act as mediating variables between willingness to donate blood and exposure to information.

## Data availability statement

The original contributions presented in the study are included in the article/Supplementary material, further inquiries can be directed to the corresponding author.

## Ethics statement

Ethical approval was not required for the study involving humans in accordance with the local legislation and institutional requirements. The studies were conducted in accordance with the local legislation and institutional requirements. Written informed consent to participate in this study was not required from the participants in accordance with the national legislation and the institutional requirements.

## Author contributions

ZZ: Writing – review & editing, Investigation. QL: Investigation, Writing – original draft.

## Footnotes

^1^ www.wjx.cn

## References

[1] Li Z Lei S Li X Zhao Y Dai Y Jin S (2021). Blood donation fear, perceived rewards, self-efficacy, and intention to return among whole blood donors in China: a social cognitive perspective. Front Psychol 12:683709. doi: 10.3389/fpsyg.2021.683709, PMID: 34880801 PMC8645584

[2] Elias E Mauka W Philemon RN Damian DJ Mahande MJ Msuya SE (2016). Knowledge, attitudes, practices, and factors associated with voluntary blood donation among university students in Kilimanjaro. Tanzania J blood transfusion 2016:8546803. doi: 10.1155/2016/8546803, PMID: 28070449 PMC5192290

[3] Gao L Wang Q (2017). Survey on knowledge, attitude and practice about blood donation among continuing medical education (CME) students in Sichuan province, China. Transfus Apher Sci 56:454–8. doi: 10.1016/j.transci.2017.05.004, PMID: 28566126

[4] Liu Y Zhu J He J (2022). Can selfies trigger social anxiety? A study on the relationship between social media selfie behavior and social anxiety in Chinese youth group. Front Psychol 13:13. doi: 10.3389/fpsyg.2022.1016538, PMID: 36457931 PMC9705971

[5] Mitchell KJ Wells M Priebe G Ybarra ML (2014). Exposure to websites that encourage self-harm and suicide: prevalence rates and association with actual thoughts of self-harm and thoughts of suicide in the United States. J Adolesc 37:1335–44. doi: 10.1016/j.adolescence.2014.09.011, PMID: 25313930

[6] Lim BC Chew KY Tay SL (2022). Understanding healthcare worker's intention to donate blood: an application of the theory of planned behaviour. Psychol Health Med 27:1184–91. doi: 10.1080/13548506.2021.1946106, PMID: 34190663

[7] McMahon R Byrne M (2008). Predicting donation among an Irish sample of donors and nondonors: extending the theory of planned behavior. Transfusion 48:321–31. doi: 10.1111/j.1537-2995.2007.01526.x, PMID: 18028275

[8] Merav BN Lena G (2011). Investigating the factors affecting blood donation among Israelis. Int Emerg Nurs 19:37–43. doi: 10.1016/j.ienj.2010.01.003, PMID: 21193166

[9] Veludo-de-Oliveira TM Alhaidari IS Yani-de-Soriano M Yousafzai SY (2017). Comparing the explanatory and predictive power of intention-based theories of personal monetary donation to charitable organizations. Voluntas 28:571–93. doi: 10.1007/s11266-016-9690-7

[10] Syed-Abdul S Fernandez-Luque L Jian W-S Li Y-C Crain S Hsu M-H (2013). Misleading health-related information promoted through video-based social media: anorexia on YouTube. J Med Internet Res 15:e30. doi: 10.2196/jmir.2237, PMID: 23406655 PMC3636813

[11] Gagne T Ghenadenik AE Abel T Frohlich KL (2018). Social inequalities in health information seeking among young adults in Montreal. Health Promot. Int. 33:390–399. doi: 10.1093/heapro/daw094 28011650

[12] Link E Baumann E (2023). A comparison of Women's and Men's web-based information-seeking behaviors about gender-related health information: web-based survey study of a stratified German sample. J Med Internet Res 25:e43897. doi: 10.2196/43897, PMID: 37195743 PMC10233438

[13] Agrawal A Tiwari AK Ahuja A Kalra R (2013). Knowledge, attitude and practices of people towards voluntary blood donation in Uttarakhand. Asian J Transfusion Sci 7:59–62. doi: 10.4103/0973-6247.106740, PMID: 23559768 PMC3613665

[14] Duh HI Dabula N (2021). Millennials' socio-psychology and blood donation intention developed from social media communications: a survey of university students. Telematics Inform 58:101534. doi: 10.1016/j.tele.2020.101534

[15] Abbasi RA Maqbool O Mushtaq M Aljohani NR Daud A Alowibdi JS (2018). Saving lives using social media: analysis of the role of twitter for personal blood donation requests and dissemination. Telematics Inform 35:892–912. doi: 10.1016/j.tele.2017.01.010

[16] Al-Hajri QR Alfayez A Alsalman D Alanezi F Alhodaib H Al-Rayes SA (2021). The impact of WhatsApp on the blood donation process in Saudi Arabia. J Blood Med 12:1003–10. doi: 10.2147/jbm.S339521, PMID: 34824556 PMC8610773

[17] Lin HC-S Lee NC-A Lu Y-C (2021). The Mitigators of ad irritation and avoidance of YouTube skippable in-stream ads: an empirical study in Taiwan. Inform 12:373. doi: 10.3390/info12090373

[18] Shah Z Chu J Ghani U Qaisar S Hassan Z (2020). Media and altruistic behaviors: the mediating role of fear of victimization in cultivation theory perspective. Int J Disaster Risk Reduction 42:101336. doi: 10.1016/j.ijdrr.2019.101336

[19] Fleisher L Bass S Ruzek SB McKeown-Conn N (2002). Relationships among internet health information use, patient behavior and self efficacy in newly diagnosed cancer patients who contact the National Cancer Institute's NCI Atlantic region Cancer information service (CIS) [research support, U.S. Gov't, P.H.S.]. Proceedings AMIA Symposium 2002:260–4. PMC2244151 12463827

[20] Ajzen I (1991). The theory of planned behavior. Organizational behavior and decision processes University of Massachusetts at Amherst 50:179–211. doi: 10.1016/0749-5978(91)90020-T

[21] Fishbein M Ajzen I (1976). Misconceptions about the Fishbein model: reflections on a study by Songer-nocks. J Exp Soc Psychol 12:579–84. doi: 10.1016/0022-1031(76)90036-6

[22] Fishbein M. Ajzen I. (1977). Belief, attitude, intention and behaviour: an introduction to theory and research. Addison-Wesley, Reading MA. Philos. Rhetor. 41:842–844. doi: 10.2307/4393175

[23] Guo Q Johnson CA Unger JB Lee L Xie B Chou C-P (2007). Utility of the theory of reasoned action and theory of planned behavior for predicting Chinese adolescent smoking [comparative study; research support, N.I.H., extramural; research support, non-U.S. Gov't]. Addict Behav 32:1066–81. doi: 10.1016/j.addbeh.2006.07.015 16934414

[24] Ghasrodashti EK (2018). Explaining brand switching behavior using pull-push-mooring theory and the theory of reasoned action. J Brand Manag 25:293–304. doi: 10.1057/s41262-017-0080-2

[25] Sutton S McVey D Glanz A (1999). A comparative test of the theory of reasoned action and the theory of planned behavior in the prediction of condom use intentions in a national sample of English young people [comparative study; research support, non-U.S. Gov't]. Health Psychol 18:72–81. doi: 10.1037/0278-6133.18.1.72 9925048

[26] Busch J. The perceptions of high school teachers of HIV/AIDS education in relation to their instruction and the theory of reasoned action. Virginia Commonwealth University ProQuest Dissertations Publishing, (1996). Available at: http://gateway.isiknowledge.com/gateway/Gateway.cgi?GWVersion=2&SrcAuth=ResearchSoft&SrcApp=EndNote&DestLinkType=FullRecord&DestApp=FullRecord&KeyUT=PQDT

[27] Caputo A (2020). Comparing theoretical models for the understanding of health-risk behaviour: towards an integrative model of adolescent alcohol consumption. Europes J Psychol 16:418–36. doi: 10.5964/ejop.v16i3.2213, PMID: 33680191 PMC7909499

[28] Wang X Zuo Z Tong X Zhu Y (2022). Talk more about yourself: a data-driven extended theory of reasoned action for online health communities. Inf Technol Manag. 2022:55–75. doi: 10.1007/s10799-022-00376-6

[29] Fisher WA Kohut T Salisbury CMA Salvadori MI (2013). Understanding human papillomavirus vaccination intentions: comparative utility of the theory of reasoned action and the theory of planned behavior in vaccine target age women and men. J Sex Med 10:2455–64. doi: 10.1111/jsm.12211, PMID: 23745833

[30] Agarwal V (2014). A/H1N1 vaccine intentions in college students: an application of the theory of planned behavior. J Am Coll Heal 62:416–24. doi: 10.1080/07448481.2014.917650, PMID: 24779428

[31] Callow MA Callow DD (2021). Older Adults' behavior intentions once a COVID-19 vaccine becomes available. J Appl Gerontol 40:943–52. doi: 10.1177/07334648211019205, PMID: 34036821

[32] Wang Y Qiao T Liu C (2023). A study of reasons for self-disclosure on social media among Chinese COVID-19 patients: based on the theory of planned behavior model. Healthcare 11:229–66. doi: 10.3390/healthcare11101509, PMID: 37239795 PMC10218332

[33] Chauhan R Kumar R Thakur S (2018). A study to assess the knowledge, attitude, and practices about blood donation among medical students of a medical college in North India. J Family Med Prim Care 7:693–7. doi: 10.4103/jfmpc.jfmpc_54_17, PMID: 30234039 PMC6132011

[34] Masser BM Hyde MK Ferguson E (2020). Exploring predictors of Australian community members' blood donation intentions and blood donation-related behavior during theCOVID-19 pandemic. Transfusion 60:2907–17. doi: 10.1111/trf.16067 32905630

[35] Liu J Han H (2023). Applying a modified and extended theory of planned behavior to predict blood donation intentions among Chinese university students: an empirical investigation. Heliyon 9:e18851. doi: 10.1016/j.heliyon.2023.e18851, PMID: 37576329 PMC10412828

[36] Chou KL (1998). Effects of age, gender, and participation in volunteer activities on the altruistic behavior of Chinese adolescents. J Genet Psychol 159:195–201. doi: 10.1080/00221329809596145, PMID: 9595702

[37] Sojka BN Sojka P (2008). The blood donation experience: self-reported motives and obstacles for donating blood. Vox Sang 94:56–63. doi: 10.1111/j.1423-0410.2007.00990.x, PMID: 18171329

[38] Mousavi F Tavabi AA Golestan B Ammar-Saeedi E Kashani H Tabatabaei R (2011). Knowledge, attitude and practice towards blood donation in Iranian population. Transfus Med 21:308–17. doi: 10.1111/j.1365-3148.2011.01080.x, PMID: 21696474

[39] Bandura A (1977). Self-efficacy: toward a unifying theory of behavioral change [research support, U.S. Gov't, P.H.S.]. Psychol Rev 84:191–215. doi: 10.1037/0033-295x.84.2.191, PMID: 847061

[40] Weimer AA Dowds SJP Fabricius WV Sthwanenflugel PJ Suh GW (2017). Development of constructivist theory of mind from middle childhood to early adulthood and its relation to social cognition and behavior. J Exp Child Psychol 154:28–45. doi: 10.1016/j.jecp.2016.10.002, PMID: 27821294 PMC5154876

[41] Al-Hchaim MHS Hermis AH Al-Mamoori HMK Jawad JM Al-Ashour IA (2022). Self-efficiency of patients with diabetes on self-care at Al-Najaf Center for Diabetes and Endocrine disorders, Babylon, Iraq. Rawal Medical J 47:806–10.

[42] Liu Y Ding X Jiang Y Zhang C Mao D Shen Z (2015). Analysis of health self-management for diabetes self-efficacy. Zhong nan da xue xue bao. Yi xue ban= J Central South University Medical Sci 40:886–90. doi: 10.11817/j.issn.1672-7347.2015.08.010, PMID: 26333497

[43] Mehta P Sharma M Lee RC (2013). Designing and evaluating a health belief model-based intervention to increase intent of HPV vaccination among college males [; randomized controlled trial]. Int Q Community Health Educ 34:101–17. doi: 10.2190/IQ.34.1.h, PMID: 24366025

[44] Ziemer KS Hoffman MA (2013). Beliefs and attitudes regarding human papillomavirus vaccination among college-age women. J Health Psychol 18:1360–70. doi: 10.1177/1359105312462432, PMID: 23188917

[45] Clowes R Masser BM (2012). Right here, right now: the impact of the blood donation context on anxiety, attitudes, subjective norms, self-efficacy, and intention to donate blood. Transfusion 52:1560–5. doi: 10.1111/j.1537-2995.2011.03486.x, PMID: 22188546

[46] Armitage CJ Reidy JG (2008). Use of mental simulations to change theory of planned behaviour variables [randomized controlled trial]. Br J Health Psychol 13:513–24. doi: 10.1348/135910707x227088, PMID: 17650364

[47] Robinson NG Masser BM White KM Hyde MK Terry DJ (2008). Predicting intentions to donate blood among nondonors in Australia: an extended theory of planned behavior. Transfusion 48:2559–67. doi: 10.1111/j.1537-2995.2008.01904.x, PMID: 18717776

[48] Zucoloto ML Goncalez T Menezes NP McFarland W Custer B Martinez EZ (2019). Fear of blood, injections and fainting as barriers to blood donation in Brazil. Vox Sang 114:38–46. doi: 10.1111/vox.12728, PMID: 30485453

[49] Lam M Masser BM Dixson BJW (2021). A branded bandage is worth a thousand words: blood branded bandages signal men's generosity and morality. Vox Sang 116:388–96. doi: 10.1111/vox.13018, PMID: 33104242

[50] Blood Donation Law of the People's Republic of China. Available at: www.gov.cn (Accessed August 25, 2023).

[51] "Medium and Long-term Youth Development Plan (2016-2025)" (2017). Chinese Government Website: www.gov.cn (Accessed August 25, 2023).

[52] Mars B Heron J Biddle L Donovan JL Holley R Piper M (2015). Exposure to, and searching for, information about suicide and self-harm on the internet: prevalence and predictors in a population based cohort of young adults. J Affect Disord 185:239–45. doi: 10.1016/j.jad.2015.06.001, PMID: 26150198 PMC4550475

[53] Pan S Zhang D Zhang J (2020). Caught in the crossfire: how contradictory information and norms on social media influence young Women's intentions to receive HPV vaccination in the United States and China. Front Psychol 11:11. doi: 10.3389/fpsyg.2020.548365 33343438 PMC7744687

[54] Armitage CJ Conner M (2001). Social cognitive determinants of blood donation. J Appl Soc Psychol 31:1431–57. doi: 10.1111/j.1559-1816.2001.tb02681.x

[55] Masser BM White KM Hyde MK Terry DJ Robinson NG (2009). Predicting blood donation intentions and behavior among Australian blood donors: testing an extended theory of planned behavior model. Transfusion 49:320–9. doi: 10.1111/j.1537-2995.2008.01981.x, PMID: 19040598

[56] Martin-Santana JD Robaina-Calderin L Reinares-Lara E Romero-Dominguez L (2019). Knowing the blood nondonor to activate behaviour. Soc Sci-Basel 8:324. doi: 10.3390/socsci8120324

[57] Kowalsky JM France CR France JL Whitehouse EA Himawan LK (2014). Blood donation fears inventory: development and validation of a measure of fear specific to the blood donation setting. Transfus Apher Sci 51:146–51. doi: 10.1016/j.transci.2014.07.007, PMID: 25151096

[58] Fornell C Larcker DF (1981). Evaluating structural equation models with unobservable variables and measurement error. J Mark Res 18:39–50. doi: 10.1177/002224378101800104

[59] Ortiz RR Shafer A Cates J Coyne-Beasley T (2018). Development and evaluation of a social media health intervention to improve Adolescents' knowledge about and vaccination against the human papillomavirus. Global pediatric health 5:2333794X18777918. doi: 10.1177/2333794x18777918, PMID: 29872667 PMC5977424

[60] Soylu D Soylu A Sen S (2022). Two significant concepts in organ donation: empathic tendency and altruism. Transpl Immunol 75:75. doi: 10.1016/j.trim.2022.101731, PMID: 36374740

[61] Crano WD Prislin R. (ed.). Attitudes and attitude change. New York: Psychology Press (2008).

[62] Martin-Santana JD Reinares-Lara E Romero-Dominguez L (2020). Modelling the role of anticipated emotions in blood donor behaviour: a cross-sectional study. J Econ Psychol 81:102325. doi: 10.1016/j.joep.2020.102325

[63] Mustafa MM Abdelfattah EN Al Rukban MO (2015). Attitude towards blood donation among university students. Int J Sci Basic Appl Res 19:82–91.

[64] George P Chittem M Dwivedi R Pal C Guntupalli Y Pati S (2022). The empathy factor: the role of empathy in knowledge, attitude, and practice of organ donation in India - a Crossectional, observational study. Indian J Transplantation 16:274. doi: 10.4103/ijot.ijot_64_21

[65] Ditto B France CR (2006). Vasovagal symptoms mediate the relationship between predonation anxiety and subsequent blood donation in female volunteers. Transfusion 46:1006–10. doi: 10.1111/j.1537-2995.2006.00835.x, PMID: 16734818

[66] Chen L Zhou Y Zhang S Xiao M (2022). How anxiety relates to blood donation intention of non-donors: the roles of moral disengagement and mindfulness. J Soc Psychol 164:43–58. doi: 10.1080/00224545.2021.2024121, PMID: 35152848

[67] Martin-Santana JD Melian-Alzola L (2022). The influence of service quality and anticipated emotions on donor loyalty: an empirical analysis in blood centres in Spain. Health Care Manag Sci 25:623–48. doi: 10.1007/s10729-022-09600-9, PMID: 35841450 PMC9674737

[68] Holly CD Torbit L Ditto B (2009). Vasovagal syncope and applied tension in the blood donation setting: the role of anxiety [meeting abstract]. Psychophysiology 46:S114–4.

[69] Evans R Ferguson E (2014). Defining and measuring blood donor altruism: a theoretical approach from biology, economics and psychology. Vox Sang 106:118–26. doi: 10.1111/vox.12080, PMID: 24117697 PMC4171832

[70] Scholz C (2010). Generation Y and blood donation: the impact of altruistic help in a Darwiportunistic scenario. Transfus Med Hemother 37:195–202. doi: 10.1159/000318023, PMID: 21048826 PMC2928841

[71] France CR France JL Himawan LK Lux P McCullough J (2021). Donation related fears predict vasovagal reactions and donor attrition among high school donors. Transfusion 61:102–7. doi: 10.1111/trf.16099, PMID: 32997822

[72] Hyde M Chambers S. Information sources, donation knowledge and attitudes towards transplant recipients in Australia. In: Transplantation. Walnut St, Philadelphia, PA, USA: Lippincott Williams & Wilkins (2013). 96:S223. 10.7182/pit2014799 24919734

[73] Nisbett RE Peng K Choi I Norenzayan A (2001). Culture and systems of thought: holistic versus analytic cognition. Psychol Rev 108:291–310. doi: 10.1037/0033-295X.108.2.291, PMID: 11381831

[74] Schumann F Steinborn MB Kürten J Cao L Händel BF Huestegge L (2022). Restoration of attention by rest in a multitasking world: theory, methodology, and empirical evidence. Front Psychol 13:867978. doi: 10.3389/fpsyg.2022.867978, PMID: 35432083 PMC9010884

[75] Lemmens KP Abraham C Ruiter RA Veldhuizen IJ Dehing CJ Bos AE (2009). Modelling antecedents of blood donation motivation among non-donors of varying age and education. Br J Psychol 100:71–90. doi: 10.1348/000712608X310237, PMID: 18547458

