# Supplementary material for: Rational or altruistic: the impact of social media information exposure on Chinese youth’s willingness to donate blood
Source: Front Public Health. 2024 May 9;12:1359362. doi: 10.3389/fpubh.2024.1359362 (PMC11112013; doi:10.3389/fpubh.2024.1359362)
Supplement: Supplementary file 1 [file Data_Sheet_1.docx]

Supplementary Material

# Supplementary Data

Supplementary Material should be uploaded separately on submission. Please include any supplementary data, figures and/or tables.

Supplementary material is not typeset so please ensure that all information is clearly presented, the appropriate caption is included in the file and not in the manuscript, and that the style conforms to the rest of the article.

# Supplementary Figures and Tables

## Supplementary Figures

**Supplementary Figure 1**

**
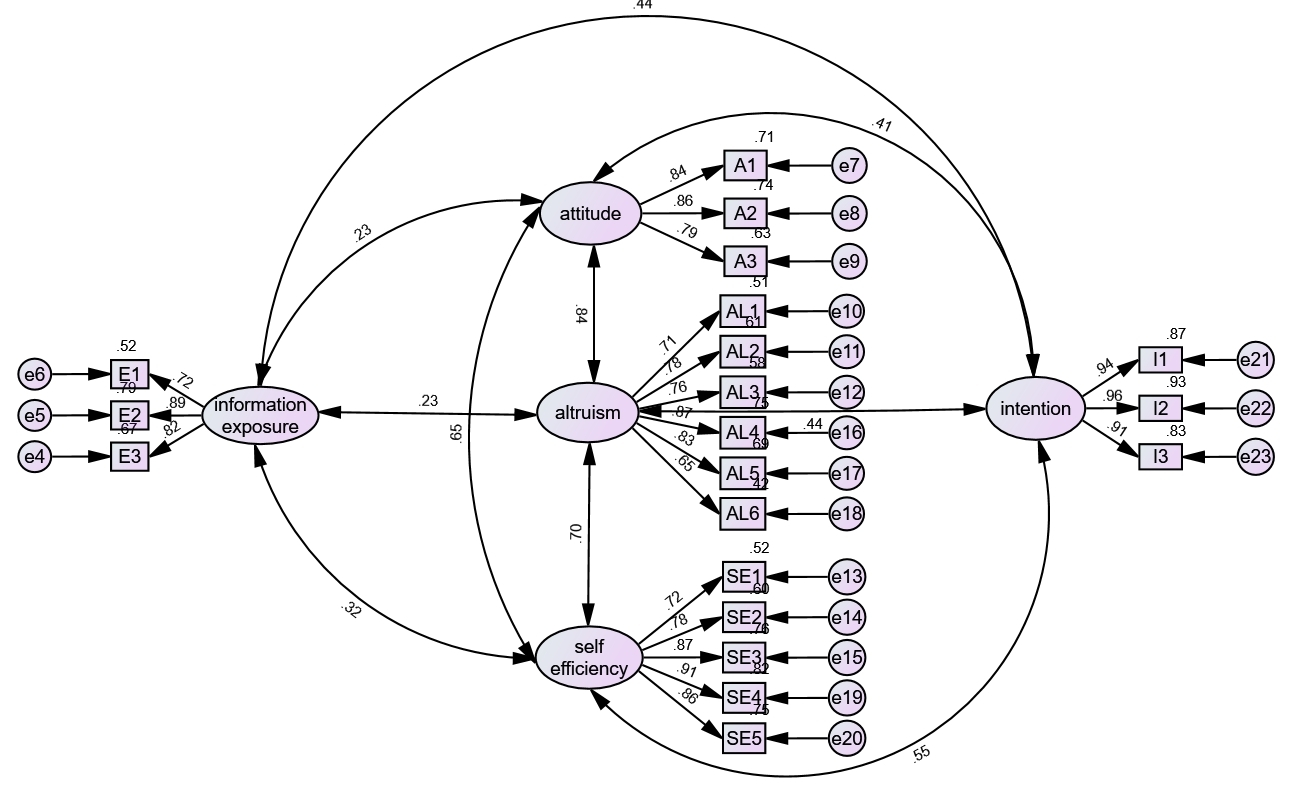
**

**Supplementary Figure 2**

**
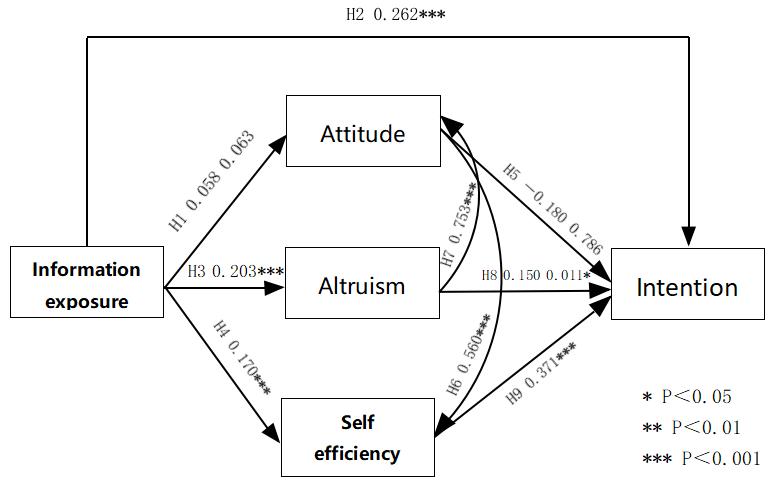
**

## 2.2Supplementary Tables

| **TABLE 1** Demographic characteristics of participants and Differential testing of demographic characteristics in behavior (N=455) | | | | | | | |
| --- | --- | --- | --- | --- | --- | --- | --- |
| **Characteristic** | **Demographic information** | **Frequency** | **%** | **Mean** | **SD** | **t, F, or r** | **P-value** |
| Gender* | Male | 134 | 29.4 | 1.71 | 0.456 | 0.247 | 0.769 |
|  | Female | 321 | 70.6 |  |  |  |  |
| Age | 18-24 | 269 | 59.1 | 2.41 | 0.492 | 2.491 | 0.115 |
|  | 25-34 | 186 | 40.9 |  |  |  |  |
| Education | Primary school and below | 2 | 0.4 | 4.45 | 0.634 | 0.243 | 0.962 |
|  | Junior High School | 2 | 0.4 |  |  |  |  |
|  | Senior High School | 5 | 1.1 |  |  |  |  |
|  | Bachelor college | 236 | 51.9 |  |  |  |  |
|  | Master's degree | 200 | 44 |  |  |  |  |
|  | Doctoral degree and above | 10 | 2.2 |  |  |  |  |
| Monthly Income  (CNY) | <3000 | 255 | 56 | 1.77 | 1.01 | 0.632 | 0.595 |
|  | 3000-5999 | 93 | 20.4 |  |  |  |  |
|  | 6000-9999 | 65 | 14.3 |  |  |  |  |
|  | ≥10000 | 42 | 9.3 |  |  |  |  |
| Variables with "*" indicate the heterogeneity of variance, using the Welch method.  SD is the standard deviation.  "CNY" refers to the China Yuan. | | | | | | | |

| TABLE 2 Correlation analysis between different variables | | | | | | | |
| --- | --- | --- | --- | --- | --- | --- | --- |
|  | Information Exposure | Altruism | Attitude | Self-efficiency | Intention | M | SD |
| Information Exposure | 1 |  |  |  |  | 2.739 | 1.130 |
| Altruism | .203** | 1 |  |  |  | 4.003 | 0.764 |
| Attitude | .210** | .762** | 1 |  |  | 3.344 | 0.826 |
| Self-efficiency | .390** | .673** | .596** | 1 |  | 4.807 | 1.332 |
| Intention | .288** | .434** | .368** | .530** | 1 | 2.531 | 1.24876 |
| **P＜0.01 | | | | | | | |

| TABLE 3 Factor loadings | | | | | | |
| --- | --- | --- | --- | --- | --- | --- |
| Variables | Item 1 | Item 2 | Item 3 | Item 4 | Item 5 | Item 6 |
| Information exposure | 0.725 | 0.889 | 0.818 | / | / | / |
| Intention | 0.935 | 0.964 | 0.909 | / | / | / |
| Attitude | 0.836 | 0.878 | 0.774 | / | / | / |
| Self-efficiency | 0.734 | 0.814 | 0.891 | 0.865 | 0.812 | / |
| Altruism | 0.722 | 0.794 | 0.77 | 0.868 | 0.83 | 0.879 |

| TABLE 4 Reliability and convergence validity testing results | | | |
| --- | --- | --- | --- |
| Latent variables | Cronbach's Alpha | AVE | CR |
| Information exposure | 0.852 | 0.662 | 0.854 |
| Intention | 0.955 | 0.877 | 0.955 |
| Attitude | 0.868 | 0.690 | 0.869 |
| Self-efficiency | 0.913 | 0.681 | 0.914 |
| Altruism | 0.886 | 0.660 | 0.921 |
| CR is the composite reliability; AVE is the average variance extracted, Cronbach's Alpha is the extent to which the items represent the scale. | | | |

| TABLE 5 Discriminant validity testing results | | | | | | | |
| --- | --- | --- | --- | --- | --- | --- | --- |
|  | Information Exposure | Altruism | Attitude | Self-  efficiency | Intention | |  |
| Information Exposure | 0.813 |  |  |  |  | |  |
| Altruism | 0.208*** | 0.812 |  |  |  | |  |
| Attitude | 0.230*** | 0.787*** | 0.830 |  |  | |  |
| Self-efficiency | 0.321*** | 0.688*** | 0.653*** | 0.829 |  | |  |
| Intention | 0.437*** | 0.467*** | 0.414*** | 0.562*** | 0.936 | |  |
| The bold values indicate the square root of AVE | | | | | |  |  |

| TABLE 6 Model fitting indexes after modification | | | | | |  |
| --- | --- | --- | --- | --- | --- | --- |
| Index | x2/df | RMSEA | CFI | TLI | NFI |  |
| Observed value | 3.176 | 0.069 | 0.952 | 0.942 | 0.914 |  |
| Ideal value | ＜5 | ＜0.08 | ＞0.9 | ＞0.9 | ＞0.9 |  |
| RMSEA is the root means square error of approximation;  NFl.Normed fit index;  TLI.Tucker-Lewis index:  CFl.comparative fit index. | | | | | | |

| TABLE 7. Hypothesis testing results | | | | |
| --- | --- | --- | --- | --- |
| Hypothesis | Model Paths | Path coefficients(β) | P-values | Results |
| H1 | Information exposure→Attitude | 0.058 | 0.063 | Not supported |
| H2 | Information exposure →Intention | 0.262 | *** | Supported |
| H3 | Information exposure→Altruism | 0.203 | *** | Supported |
| H4 | Information exposure→Self-efficiency | 0.170 | *** | Supported |
| H5 | Attitude→ Intention | -0.180 | 0.786 | Not supported |
| H6 | Attitude→Self-efficiency | 0.560 | *** | Supported |
| H7 | Altruism→Attitude | 0.753 | *** | Supported |
| H8 | Altruism→Intention | 0.150 | 0.011* | Supported |
| H9 | Self-efficiency→Intention | 0.371 | *** | Supported |
